# IndoorPlant: A Model for Intelligent Services in Indoor Agriculture Based on Context Histories

**DOI:** 10.3390/s21051631

**Published:** 2021-02-26

**Authors:** Bruno Guilherme Martini, Gilson Augusto Helfer, Jorge Luis Victória Barbosa, Regina Célia Espinosa Modolo, Marcio Rosa da Silva, Rodrigo Marques de Figueiredo, André Sales Mendes, Luís Augusto Silva, Valderi Reis Quietinho Leithardt

**Affiliations:** 1 Applied Computing Graduate Program, University of Vale do Rio dos Sinos, Av. Unisinos 950, Bairro Cristo Rei, São Leopoldo, RS 93022-750, Brazil; ghelfer@edu.unisinos.br; 2 Electrical Engineering Graduate Program, University of Vale do Rio dos Sinos, Av. Unisinos 950, Bairro Cristo Rei, São Leopoldo, RS 93022-750, Brazil; mmrsva@gmail.com (M.R.d.S.); marquesf@unisinos.br (R.M.d.F.); 3 Agronomic Engineering Undergraduate Program, University of Vale do Rio dos Sinos, Av. Unisinos 950, Bairro Cristo Rei, São Leopoldo, RS 93022-750, Brazil; reginaem@unisinos.br; 4 Expert Systems and Applications Lab—ESALAB, Faculty of Science, University of Salamanca, Plaza de los Caídos s/n, 37008 Salamanca, Spain; andremendes@usal.es (A.S.M.); luisaugustos@usal.es (L.A.S.); 5 Laboratory of Embedded and Distribution Systems, University of Vale do Itajaí, Rua Uruguai 458, C.P. 360, Itajaí, SC 88302-901, Brazil; 6 VALORIZA, Research Center for Endogenous Resources Valorization, Instituto Politécnico de Portalegre, 7300-555 Portalegre, Portugal; valderi@ipportalegre.pt

**Keywords:** computing in agriculture, indoor agriculture, prediction in agriculture, context awareness in agriculture, context histories in agriculture

## Abstract

The application of ubiquitous computing has increased in recent years, especially due to the development of technologies such as mobile computing, more accurate sensors, and specific protocols for the Internet of Things (IoT). One of the trends in this area of research is the use of context awareness. In agriculture, the context involves the environment, for example, the conditions found inside a greenhouse. Recently, a series of studies have proposed the use of sensors to monitor production and/or the use of cameras to obtain information about cultivation, providing data, reminders, and alerts to farmers. This article proposes a computational model for indoor agriculture called IndoorPlant. The model uses the analysis of context histories to provide intelligent generic services, such as predicting productivity, indicating problems that cultivation may suffer, and giving suggestions for improvements in greenhouse parameters. IndoorPlant was tested in three scenarios of the daily life of farmers with hydroponic production data that were obtained during seven months of cultivation of radicchio, lettuce, and arugula. Finally, the article presents the results obtained through intelligent services that use context histories. The scenarios used services to recommend improvements in cultivation, profiles and, finally, prediction of the cultivation time of radicchio, lettuce, and arugula using the partial least squares (PLS) regression technique. The prediction results were relevant since the following values were obtained: 0.96 (R^2^, coefficient of determination), 1.06 (RMSEC, square root of the mean square error of calibration), and 1.94 (RMSECV, square root of the mean square error of cross validation) for radicchio; 0.95 (R^2^), 1.37 (RMSEC), and 3.31 (RMSECV) for lettuce; 0.93 (R^2^), 1.10 (RMSEC), and 1.89 (RMSECV) for arugula. Eight farmers with different functions on the farm filled out a survey based on the technology acceptance model (TAM). The results showed 92% acceptance regarding utility and 98% acceptance for ease of use.

## 1. Introduction

Technological advances have been expanding the modernization of agriculture and, as a result, increasing the productivity and immunity of planted crops. A factor that generates the search for increased agricultural productivity is that the agricultural sector consumes approximately 70% of the freshwater available [1]. Studies indicate that due to the growth of the world population and consequently greater consumption of food, the production of food is expected to increase by 60% by the year 2050.

The demand for food needs to be met in the face of challenges such as increased climate change and environmental impacts resulting from intensive agricultural practices. Intelligent agriculture, based on Internet of Things (IoT) technologies, allows farmers to reduce waste and increase productivity, from irrigation with greater precision to the amount of fertilizer used [2]. In addition, it can help prevent pollution [3], monitoring climatic conditions at low cost [4], and bring solutions by adding artificial intelligence methods [5,6].

Agriculture is adopting technological resources designed to increase yield from planting to harvest, through monitoring, recommendations [7], and support [8]. As information related to crops is increasingly accessible, there are studies by the scientific community on context awareness [9,10] and on prediction [11,12,13]. IndoorPlant uses context histories [14,15] instead of routes. This is because context histories do not refer only to the displacement history, but to a wide variety of information obtained from the entities.

Species normally cultivated in open fields are increasingly being grown in closed environments such as greenhouses and/or pavilions. This change occurs because greenhouses provide the creation of a microclimate more favorable for the species, and thus better results are obtained in terms of the reduction of pests, less use of pesticides, and greater production, among other aspects. Therefore, the monitoring and control of the greenhouse microclimate is a real problem, where producers must deal with various parameters to ensure the ideal growth of crops [16]. This work uses the concept of Smart Farming [17], which represents the use of information and communication technology (ICT) systems applied in agriculture, leading to the Third Green Revolution [18]. One of the great differentials of Smart Farming is that it also seeks to drive new trends such as family farming.

In this scenario, the IndoorPlant model provides generic intelligent services based on context histories for users. IndoorPlant focuses on users who have greenhouse crops, and related segments such as farmers and suppliers of agricultural inputs. The prototype allowed a practical model evaluation with the creation of a bot and collection of the contexts of cultivation, to offer intelligent services through the context histories. IndoorPlant obtained the parameters of greenhouses used for hydroponic cultivation of three different species (radicchio, arugula, and lettuce) for seven months. Each greenhouse had an area of 500 m^2^ and a species planted at different levels of growth. Eight farmers with different functions used the prototype and answered a survey based on the technology acceptance model (TAM) to assess usefulness and ease of use.

The main objective of this work is to create a computing model for indoor agriculture that uses the historical context of crops and provides intelligent generic services for farmers in different types of cultivation. This article is organized into five sections. Section 2 presents work interconnected with IndoorPlant and compares them. Section 3 presents the IndoorPlant model, mainly focusing on its computational architecture and requirements. Section 4 presents the methodology for creating the prototype; the functionalities of the model are also evaluated through scenarios and, finally, a questionnaire is applied to assess farmers’ perceptions. The last section contains the considerations, conclusion, and future work.

## 2. Related Works

This section presents 11 studies related to smart agriculture that use the data obtained from sensors installed in the plantation and some prediction techniques with the information obtained. The paragraphs listed below present their objectives, differentials, discussing the state of the art, and the perceived gaps.

Santos et al. [19] presented a model that combines a wireless network range system (LoRa) with a prediction mechanism that proactively anticipates possible crop dysfunctions, notifying the farmer of corrective actions as soon as possible. The proposed model was applied in a 12 m^2^ greenhouse with soil cultivation. Goap et al. [20] proposed an intelligent system, applied in soil agriculture, programmed in open code that provides for the irrigation requirements of the field using sensors. Mehra et al. [21] developed a hydroponic system based on intelligent IoT using neural networks; the system is smart enough to control the actions of the hydroponic environment.

Alipio et al. [22] developed an intelligent hydroponics system used in automation of the crop growth process using inference through Bayesian networks. Huong et al. [23] proposed a generic model using the Markov decision model to create automatic and accurate irrigation. The model seeks to transform agriculture and be more efficient in energy and water consumption. Sisyanto et al. [8] created a system that allows farmers to monitor their hydroponics through Telegram Messenger. Ni et al. [24] considered the effects of the use of a variable spray system in Unmanned Aerial Vehicles (UAVs). The work used a data set to train support vector regression and back propagation neural network.

The technology of Plant-Microbial Fuel Cell (PMFC) [25] is found in works with similar characteristics, namely, IoT smart sensing nodes, self-sustaining, ultra-low power devices able to transmit environmental data over a long range [26,27,28]. Brunelli et al. [29] presented an ultra-low power smart camera capable of detecting and recognizing pests in an apple field using a neural networks approach. An evolution of this system used Raspberry Pi [30] and Intel Movidius Neural Compute Stick [31], both powered by a solar panel.

Table 1 compares these works and IndoorPlant according to the type of sensor they use, which plant species were cultivated, data analysis and storage, prediction technique, and use of historical contexts. The analysis in Table 1 shows that only IndoorPlant uses the information from the crops as historical contexts; the other studies only use the information to train their services. That is, IndoorPlant can make recommendations for cultivation based on previous contexts, for example, when it notes some parameters of the current context of cultivation outside the standards of previous contexts. With this, IndoorPlant tells the farmer to modify the cultivation parameter; however, the farmer needs to approve this change.

Another aspect is that the previous studies had only one specific service, such as predicting the temperature and humidity of the greenhouse [19], intelligent irrigation [23] or monitoring environment data [26,27,28,29,30,31]. IndoorPlant, on the other hand, can provide several intelligent services for the user, such as predicting harvest time, recommending improvements in cultivation, and alarms for any problem found in cultivation.

The type of cultivation is another differential between IndoorPlant and other studies: the proposed model supports different types of cultivation, such as hydroponics, fertigation (semi-hydroponics), and soil cultivation. The other studies focused on only one type of cultivation: Goap et al. [20] applied their system in soil agriculture, Alipio et al. [22] and Sisyanto et al. [8] in hydroponics.

## 3. IndoorPlant Model

This section describes the model for indoor agriculture that supports generic smart services based on background histories, called IndoorPlant. Section 3.1 presents an overview of the model and its main concepts together with its requirements. In Section 3.2, the architecture with the model components is presented.

### 3.1. Model Overview

In most cases, indoor agriculture happens in places close to large urban centers, since these places are where the greatest food consumption occurs. With this small distance between large cities and indoor plantations, it is possible to say that in most cases access to the Internet and the possibility of installing sensors is greater than in ordinary agriculture.

The IndoorPlant model focuses on indoor agriculture, providing intelligent generic services by storing and analyzing context histories. The main objective is to facilitate the work of farmers by using technology in their daily lives and generating recommendations for improving the crop. These recommendations and services may be different for each farmer due to the wide variety of technology installed today in greenhouses around the world.

The proposed model does not generate a suggestion for the farmers to “turn on ventilation” if their greenhouses do not have this technology installed. On the other hand, if the farmer has a greenhouse with the parameters monitored 24 h a day, IndoorPlant can analyze the history of contexts and generate recommendations on how to modify any parameter in the greenhouse for which a better result has been previously obtained when using a value different to the current one. IndoorPlant supports the management of semi-hydroponics, hydroponics, and even planting in the soil, but this must always occur within greenhouses or pavilions.

The following requirements for IndoorPlant specifications were defined:

- Monitor the entities (greenhouses), and present information about them;
- Allow access to the model through mobile devices (smartphones and tablets);
- Use context histories to generate generic smart services and recommendations;
- Support intelligent services for different purposes;
- Link notes provided by farmers during the harvest to current contexts;
- Generate recommendations for improvements in cultivation based on historical backgrounds and notes provided by farmers at harvest;
- Allow the integration of data external to the model, as well as offering data to other systems through a web server or local server called IndoorServer;
- Use profiles for users and for the plants being grown.

### 3.2. IndoorPlant Architecture

**Figure 1:**
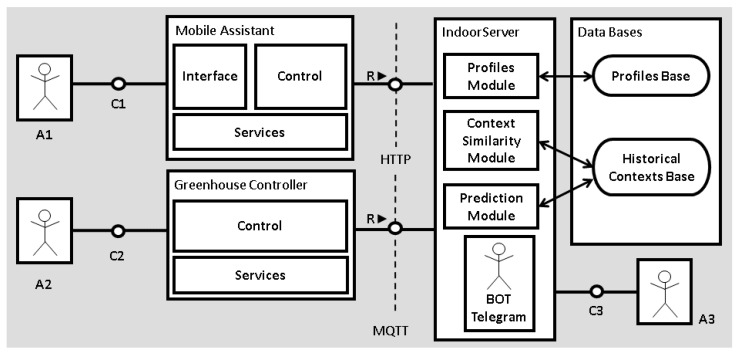
IndoorPlant architecture.

IndoorPlant utilizes the Unified Modeling Language (UML) with additional definitions proposed in the technical architecture modeling standardization [32] to create the model architecture. Figure 1 presents the architecture composed of actors (A1, A2, A3) and their accesses, and the blocks (Mobile Assistant and its components; Greenhouse Controller and its components; IndoorServer with its modules; and the Database with its bases). The communication channels are shown by the symbols C1, C2, and C3. The components appear inside the Mobile Assistant (interface, control, and services) and Greenhouse Controller (control and services) blocks and allow the actors (A1 and A2) to interact with the IndoorServer. IndoorPlant organizes the information to obtain the data generated by the three actors, and after processing the data, it provides some contextual information according to the request made. For this, IndoorPlant includes four blocks: the server called IndoorServer, the Mobile Assistant, the Greenhouse Controller, and the Databases.

The Mobile Assistant receives the actions of the mobile client (A1) through the communication channel (C1) and communicates with the server using web services, using RESTful methods to demonstrate the cultivation conditions within each greenhouse. The IndoorServer module responsible for communicating with this assistant is the Telegram bot module, created to facilitate understanding and handling by most farmers because it is in text form.

The Greenhouse Controller communicates with the server through the Message Queuing Telemetry Transport (MQTT) protocol, created specifically for use in sensors and devices in the IoT, and it also has communication methods for sending and receiving updates and new parameters. The Greenhouse Controller also collects greenhouse and greenhouse cultivation data and sends it to the IndoorServer. The controller itself acts on aspects such as maintaining the level of the reservoirs and irrigating at specific times. This crop information is processed by the context similarity module on the IndoorServer and later sent to the database called Historical Database of Contexts. The context similarity module processes information and inserts markings into the context histories as it is processed. For managing the context histories, the information is stored as presented in Table 2.

The IndoorServer component communicates with the other components through internal channels and with the External Data actor (A3), through channel C3. The IndoorServer includes mechanisms for treatment, analysis of similarity, and prediction of data regarding conditions in greenhouses. This analysis and a possible suggestion of improvements in the greenhouse parameters occur according to the information that the Greenhouse Controller passes to the server.

Within the IndoorServer, the context similarity module is responsible for processing and saving the data in its database. The function of the similarity module is to generate the user’s database by analyzing the received context histories. With this analysis, the similarity module generates a base that does not contain a history of repeated contexts and it is saved in sequence. The module compares the data received in the last receipt with the data received in the last context update, and with that, it can determine whether the data are similar or not. The data received in the last update will only be added to the database if they are not like the context of the last update.

The context similarity module processes the received data and through its rule, it always obtains a value between (0, 1) as a response; this response is the similarity score. According to Cha [33], the similarity score or similarity coefficient is the value that expresses the distance between two objects. If the answer is a value close to 0, it shows that the data received are different from the previous data, and if the answer is close to 1 it indicates that the data are similar or identical to the previous data. Along with this rule, there is a configuration that considers that if the calculated values are greater than or equal to 0.8, the contexts are similar. Among the most used distance measurements, Euclidean and Manhattan distances can be mentioned.

The peripherals that IndoorPlant uses in the Greenhouse Controller are a GSM/GPRS module (SIM900) to send information and alerts to users, a Wi-Fi module (ESP8266) to communicate with the server without using cables, an Ethernet module (ENC28J60) to communicate with the server via cables, cameras to photograph and to be able to analyze the plantation with an image processing algorithm, and a display to show information on the cultivation in place. Activations that the model can perform are: reset the level of the circulation tanks, irrigate at scheduled times, turn on the circulation pump, turn on/off lighting, open/close the shade, turn on/off the hoods, increase or decrease the number of inputs for the plants, turn on the sprinklers, and turn on refrigeration of the nutrient solution.

In addition to these suggestions for improvements, IndoorPlant has a prediction module that aims to predict production for the month based on its history of contexts, and to predict alerts before the memos are triggered by the greenhouse control system. The model does not support only these two prediction services; IndoorPlant can accept other prediction services and uses data external to the model. These external data are shown in Figure 1 as actor 3 (A3). IndoorPlant can also compare the actual production for the month, calculate what the maximum production would be according to the existing data, and inform the farmer via an application if there is any control error in the greenhouse, among other services.

This diversity in predictions occurs due to the variables provided in the context histories and the equivalent results they generate. The results that the prediction provides must have the same meaning/objective as the data provided previously. That is, if IndoorPlant receives context histories with eight variables and one variable is the quantity of strawberries harvested, the other seven variables are related to the number of strawberries harvested. IndoorPlant will also predict the number of strawberries that it will be possible to harvest with the current context of planting, if it has the same information as previously passed on. IndoorPlant cannot predict the productivity of a greenhouse if previous context histories do not have the previous information for how many strawberries were harvested.

The Profile module is responsible for creating a profile for each actor or greenhouse that is part of IndoorPlant. Every time the module receives data from a profile, it interprets the data and makes a comparison between the data already existing in the database that has the profiles, called the Profiles Base. The comparison that the module makes is to analyze if any profile already saved in the Profiles Base is different to what is being received in real time. The profile module will not add data related to the received profile if they are completely the same.

If any parameter is different and all others are the same, the module already interprets it as a different profile and saves the profile in its database. This is because if the profile is related to the control parameters of a greenhouse and all indices are the same but only the cultivation greenhouse is different, a different greenhouse already has an influence, as plants may not be arranged close to the ground and, with that, suffer the action of solar lighting differently.

IndoorPlant does not have its own forecasting tool. The tool used in the Prediction module for data analysis and cultivation time forecasting is ChemoStat, an online multivariate data analysis tool [34]. These multivariate data are usually in the form of a multivariate matrix X, where the matrix is composed of m variables for n samples. ChemoStat uses a multiple regression algorithm called partial least squares (PLS) [35,36]. PLS considers that its predictors are not fixed, that is, its predictors can be measured with error. Due to this variation, PLS becomes more robust in relation to measurement uncertainty.

As answers to the prediction, IndoorPlant uses the coefficient of determination (R^2^), square root of the mean square error of calibration (RMSEC), and the square root of the mean square error of cross validation (RMSECV). R^2^ can vary between 0 and 1, and the closer the R^2^ value is to 1, the better the agreement between the model and the sample. RMSEC presents the mean quadratic error in relation to the variable being obtained as a result, in the case of IndoorPlant, cultivation days. RMSECV, on the other hand, has the same purpose as RMSEC, but it validates the error by crossing samples, not following a line for elaboration of the error.

IndoorPlant applies the PLS model due to the linear relationship between the data and their interest properties, in addition to the use of more than one variable (pH, EC, temperature of the nutrient solution, air temperature, humidity of the air, and cultivation days). The PLS is applied in chemistry in analysis of food, drug, and fuel, but few works used this model for data correlation between sensors and crop productivity. Helfer et al. [37] used a time series of weather sensors data and wheat productivity in a PLS model with R^2^ = 0.92.

IndoorPlant proposes an ontology for indoor agriculture to manage three aspects: the profile of the people who use the system, the parameters of the greenhouses, and the characteristics of each cultivated species. This ontology is called Agrindoor and was developed using the Protégé tool. An ontology provides standardization of information, assisting in the exchange of messages, in the visualization of terms, and in storage. Ontology manages the domain of the people indicated in the use cases above, greenhouse parameters, reading of sensors, and the technical specifications of plants.

**Figure 2:**
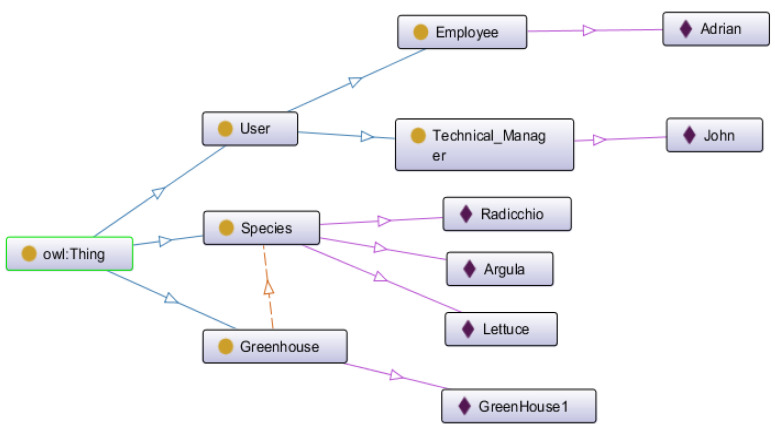
Agrindoor ontology and classes, with some examples.

Figure 2 shows the IndoorPlant classes without showing all instances for each class; only some instances of the classes are presented to exemplify its use. In addition to the User class and its subclasses (Technical Manager and Employee), the ontology for the Greenhouse class was also created, which has the capacity to store the greenhouse conditions as data properties. Finally, the Species class ontology was created with the characteristics of the plants also being inserted as data properties.

## 4. Evaluation Methodology

This section describes the prototype and details the evaluation. Section 4.1 describes the technologies used in development of the prototype; Section 4.2 presents information related to the model evaluation. The evaluation took place in three test scenarios, composed of real data from hydroponic cultivation data sets. A questionnaire was also applied based on the concepts of TAM. The final considerations of the section are presented in Section 4.3.

### 4.1. Prototype

**Figure 3:**
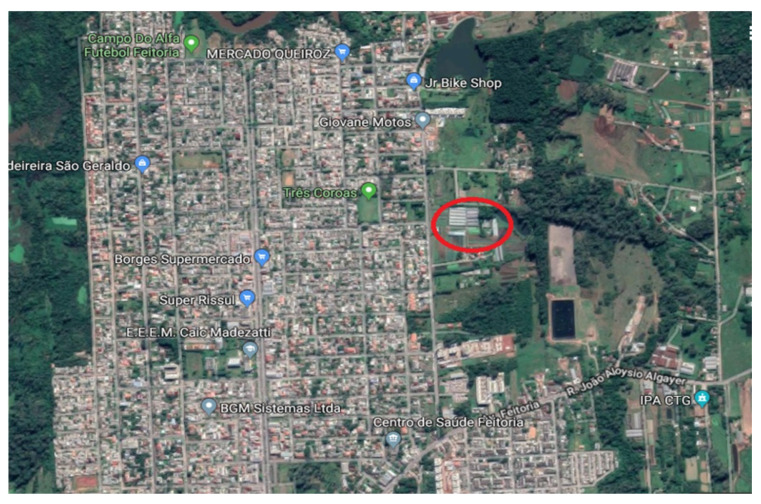
Location of farm that tested IndoorPlant.

The IndoorServer prototype uses Node-RED software [38]. The communication protocol between the IndoorServer and the Greenhouse Controller was MQTT. The prototype was installed in an olive growing company, located in the city of São Leopoldo/RS, Brazil. Figure 3 shows the location of the property (red circle) and shows the proximity of the rural property to the city. To collect data on hydroponic cultivation, the Greenhouse Controller used was Cultiva Fácil Hidroponia, a system developed by the company BGM Sistemas Ltd., also from São Leopoldo/RS.

**Figure 4:**
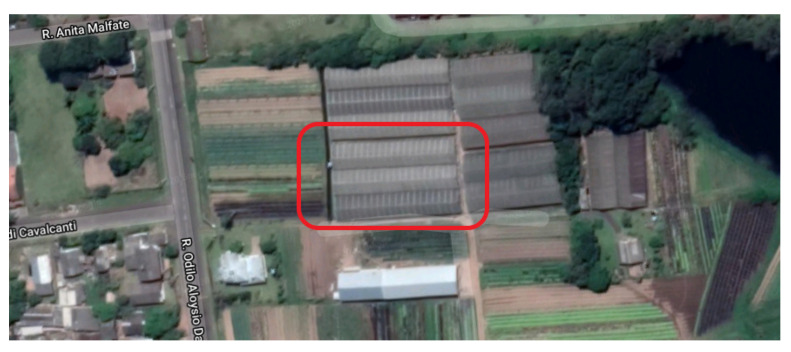
Aerial image of the greenhouses that used the prototype.

**Figure 5:**
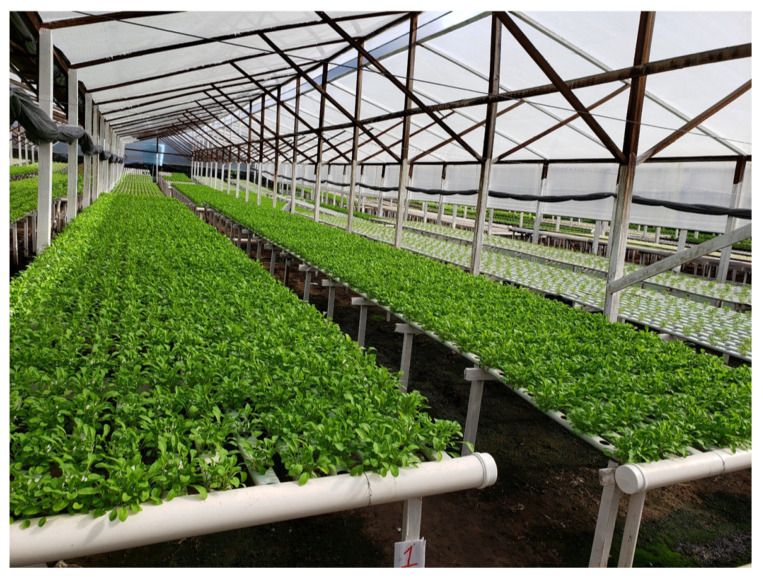
Internal image of greenhouses with hydroponic cultivation.

Data were collected in three hydroponic greenhouses, each with an area of 500 m^2^. Figure 4 shows the distribution of the greenhouses. Each greenhouse had a different plant culture: lettuce, arugula, or radicchio crops. Figure 5 presents an internal view of the facilities, with hydroponic cultivation present throughout the greenhouse. The database used on the IndoorServer was MongoDB; two databases were created according to the publication pattern in the model architecture in Figure 1. A database of records and a database of historical contexts were created. MongoDB was chosen because it is scalable, has high performance, and can orient objects. This choice must also be made with context data that are stored with different information, for each type of cultivation or greenhouse.

This distinction in the information is related to the type of cultivation and the technology installed in the greenhouses, as IndoorPlant does not support only one type of cultivation, and this diversity in the cultures generates different base parameters for each one. Different parameters can be seen for hydroponics and drip irrigation. In hydroponics, the most important parameters are pH, electrical conductivity, and the temperature of the solution. In dripping, the main parameters are soil moisture, temperature, and ambient humidity.

It is possible to find greenhouses with all manual control up to fully automated greenhouses. This greenhouse control is related to the microclimate conditions, the amount of irrigation, and opening and closing of greenhouses, among other factors. Due to this variation in technology, different information is also generated even with the same type of cultivation.

In addition to the Greenhouse Controller having a siren that is activated when a crop parameter reaches the limit or a problem occurs in the greenhouse, a bot was also developed next to the IndoorServer that informs farmers on their smartphones. This bot was developed within Node-RED [38] and uses the Telegram Messenger application to inform the farmer about crop suggestions, problems, and alerts. Among the possible information that IndoorPlant can send to the farmer are indices that have exceeded the limit and current indices for greenhouses. Farmers can also request information about their cultivation and/or greenhouse in real time and add notes regarding the harvest from a specific greenhouse.

The notes that the farmer gives for the harvest help IndoorPlant afterward to indicate which are the best parameters for each cultivated crop. IndoorPlant considers the parameters for the plants that were cultivated together with the notes provided by the farmer at harvest time. Because of this, IndoorPlant uses the histories of ancient contexts together with the notes provided by farmers to indicate which are the best parameters for the cultivation of a given plant.

### 4.2. Evaluation

All evaluations performed with IndoorPlant used the Greenhouse Controller (Cultiva Fácil Hidroponia) together with the IndoorServer, with the features previously mentioned. The assessments were designed to prove the model’s contributions using background histories to provide intelligent services. After obtaining the data set for the hydroponic cultivation of three cultures, it was possible to begin testing the similarity modules for historical contexts, profiles, and predictions. To validate the functionality of the three modules, three model evaluation scenarios were created using the services of recommendations, profiles, and productivity prediction.

#### 4.2.1. Scenario 1: Context Similarity Module

**Figure 6:**
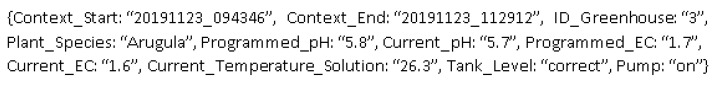
Sample of data sent for the background of historical contexts.

The first assessment scenario was built based on the data set with the historical hydroponic cultivation contexts presented above. This data set was created with data received from Cultiva Fácil Hidroponia. After data were received by the IndoorServer they were processed by the context similarity module to check if they were similar or not and subsequently sent to the context history base. As the contexts received from the Greenhouse Controller did not have exactly all the information planned in Table 2, some of them were changed so that the contexts had as many variables as possible, as shown in Figure 6.

**Figure 7:**
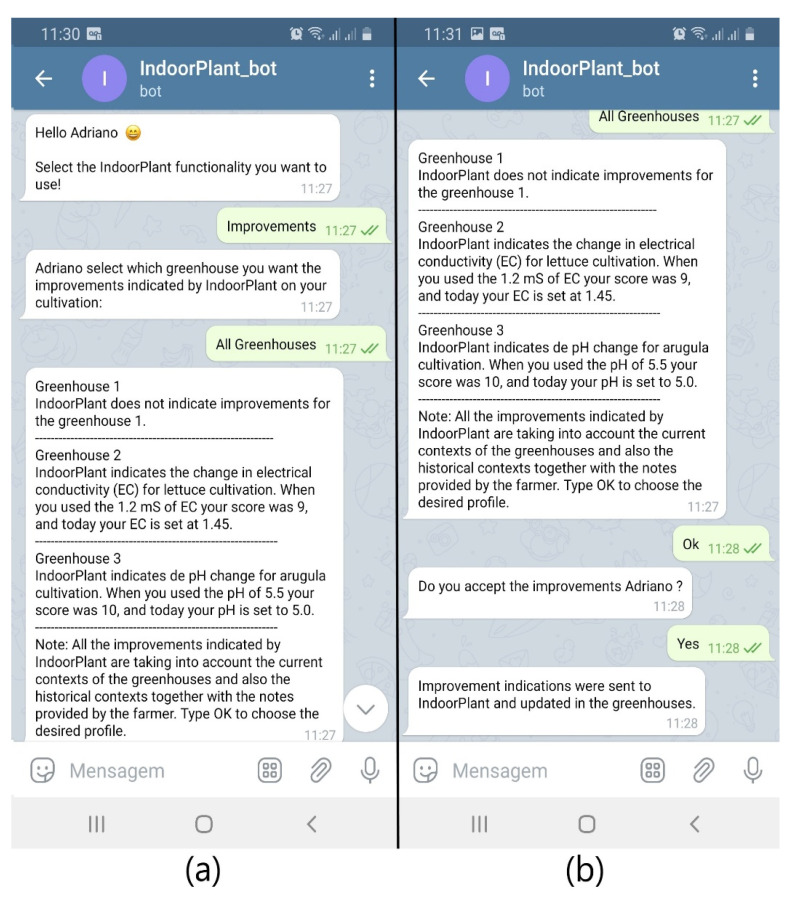
Improvements suggested by IndoorPlant (**a**) and acceptance of the suggested improvements (**b**).

This scenario presents a situation in which the farmer responsible for the cultivation (called Adriano) was traveling to a fair and the substitute farmer changed the cultivation parameters of the Greenhouse Controller without the approval of his superior. Adriano asked IndoorPlant to check the current contexts of the greenhouses; IndoorPlant found that the current contexts did not have the best parameters programmed for the context histories that obtained the best scores (Figure 7a). With this, IndoorPlant through its Telegram bot generated suggestions for adjustments to be made in each greenhouse and Adriano accepted the changes (Figure 7b).

#### 4.2.2. Scenario 2: Profiles Module

The second assessment scenario was built based on the data set with the radicchio profile data for hydroponic cultivation. The data were sent to the IndoorServer and processed by the profile module. In this module, the Euclidean and Manhattan equations are not used because only profiles that are identical to any already registered are not saved.

**Figure 8:**
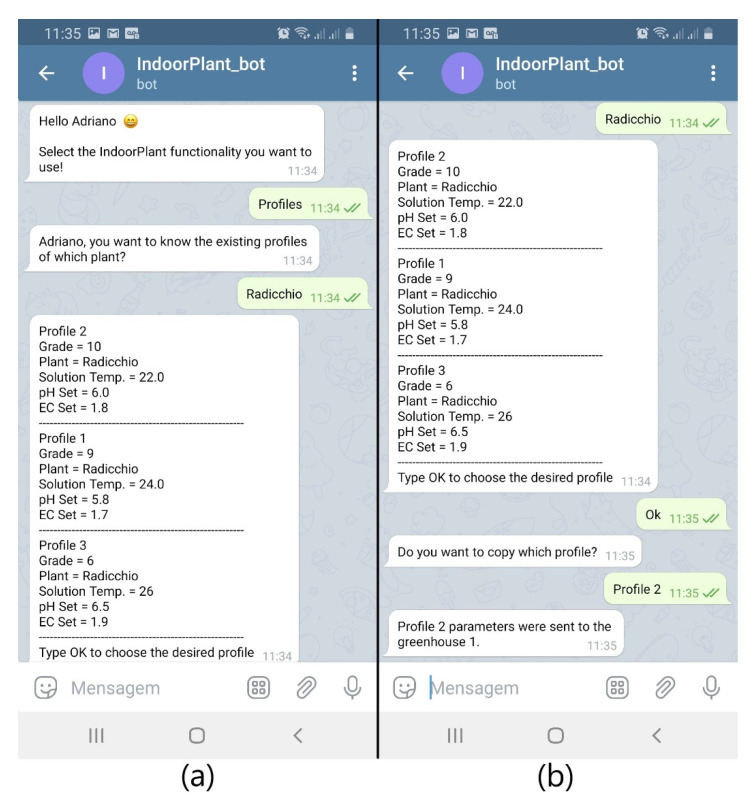
Existing (**a**) and chosen (**b**) radicchio profiles.

If the farmer plants new seedlings and wishes to use a profile they already know, they select the plant and IndoorPlant provides the existing profiles for that species (Figure 8) in descending order for the notes provided at harvest time. The farmer will then be able to freely choose which profile they want IndoorPlant to use and its parameters are sent to the Greenhouse Controller.

#### 4.2.3. Scenario 3: Prediction Module

**Figure 9:**
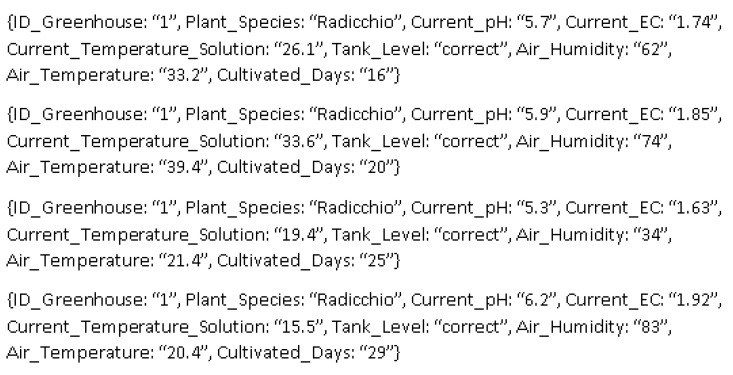
Sample of contexts related to cultivation time.

The third assessment scenario was built based on the data set with data from the historical context base along with the cultivation time for each plant. Figure 9 shows a sample of the contexts obtained for the cultivation of radicchio. This cultivation time from planting to harvest was provided by the farmer himself. The database was exported from MongoDB in CSV format and the cultivation times were added. With this information, IndoorPlant together with the prediction tool can predict the cultivation time of the planted crops.

To generate the productivity calibration model for the three crops, variables were used with the context histories of the three greenhouses for seven months of cultivation, as shown in Figure 9. Each greenhouse had its calibration model generated individually because each culture has different cultivation rates and indices, making the model more accurate for each crop. The training was done with data from the seven months of cultivation, separately for each greenhouse because they are used for different plants. With this, it is possible to know the cultivation time for each plant. It is important to note that the prediction by ChemoStat is not automated with IndoorPlant, so it was trained with seven months of cultivation, and the prediction with the current context is updated whenever necessary. In this scenario, the model used the current context in relation to the training done a week earlier. During the seven months of cultivation, the production time had significant variations. Table 3 shows the shortest and the longest time for the cultivation of each of the three plant species, in addition to the estimated and actual time of cultivation.

**Figure 10:**
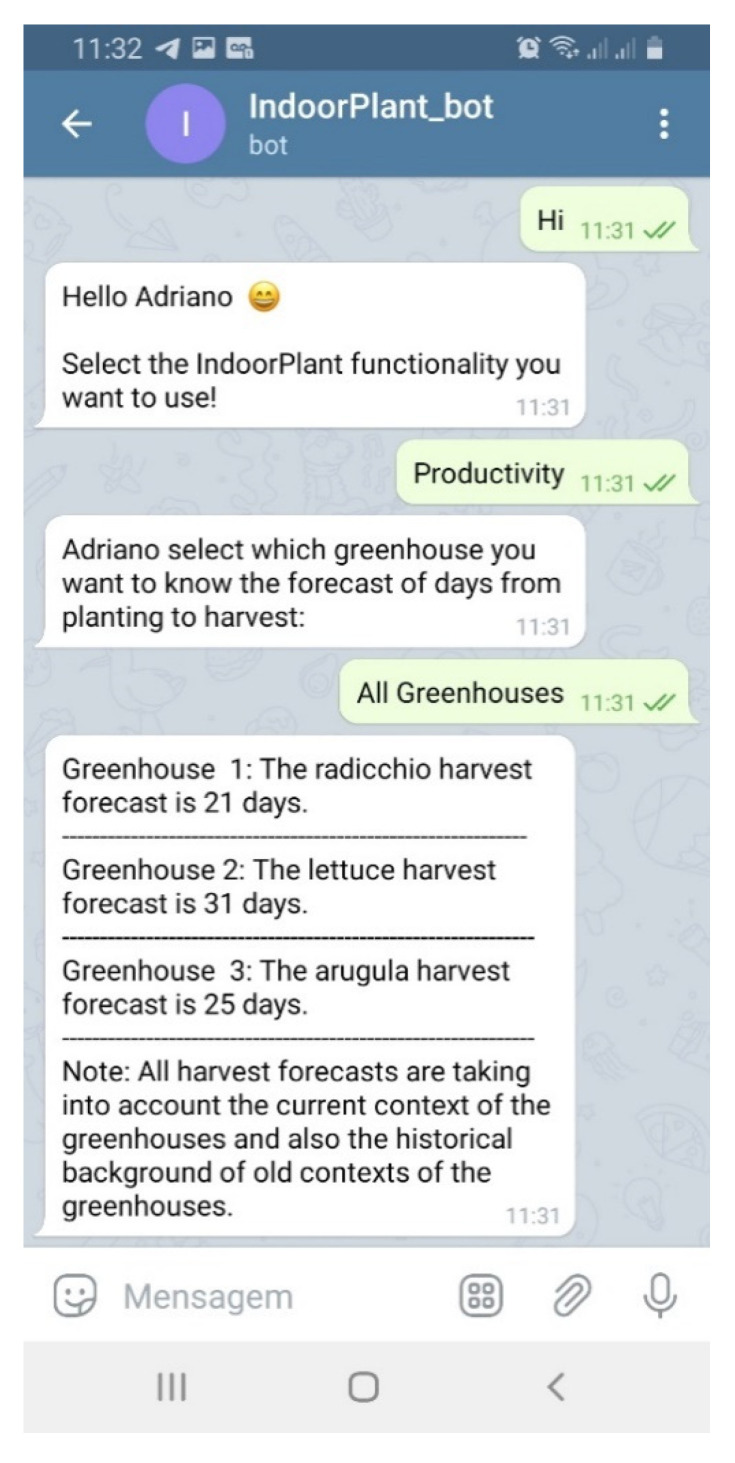
Bot menu and productivity prediction for the three plant species.

This scenario presents a situation in which the farmer responsible for stock management needs to know how long it takes for plants to reach the appropriate size for sale. Farmers need to schedule their sales so that the plants are appropriately sized on delivery days (Figure 10). Table 4 shows the forecast results for the harvest time of the three plant species. As previously mentioned, no studies were found in the scientific community that aim to predict harvest time. There are studies that predict the temperature and humidity of the greenhouse [19] and the right time to irrigate the plants [23]. This makes it difficult to compare the results obtained by IndoorPlant with those of any other study. However, as we can see in Table 4, the R^2^ rates were high. The lowest of these indices was 0.93, which indicates that we had more than 93% variance of the dependent variable from the independent variables included in the linear model.

RMSEC and RMSECV also had attractive values and show their efficiency when comparing the actual days with the expected days for the prediction of cultivation time in Table 3. As we can see, the expected time for radicchio was 21 days and the real time to harvest was 20 days. For lettuce, the expected time and real time were the same, 31 days. Finally, the expected time for arugula was 25 days and in fact it was harvested at 26 days.

Among the situations that may have influenced this difference, we can mention: the greenhouses do not have humidity, temperature, and light control and these three indices affect productivity; the tanks containing the nutrient solutions may have been cleaned during the growing season of the compared crops, which would cause a variation in the plant nutrient solution until the reservoir was filled; the seedlings when transplanted could also be minimally larger or smaller than the standard and this also influences the cultivation time; and other situations that only really appear in the day-to-day cultivation.

#### 4.2.4. TAM Evaluation

The IndoorPlant prototype was evaluated with farmers using the TAM proposed by Davis [39], later applied and expanded by Yoon and Kim [40]. The TAM model considers the following items as the main requirements for the acceptance of new technology:

- Perceived utility: the degree to which a person believes that the use of technology could improve their performance.
- Perceived ease of use: the degree to which a person believes that technology can reduce their efforts.

Table 5 presents the profile of the eight users that participated in the evaluation. The interviewees are farmers who used the prototype and have different functions within the agricultural company, as can also be seen in Table 5. Use of the bot by farmers occurred individually and always with follow-up for possible doubts; however, before use, farmers were instructed on how the bot worked. Farmers used the bot in situations like the three scenarios previously proposed, that is, contemplating the model.

To answer the questionnaire, the Likert scale was made available to users, with five levels: 1—strongly disagree, 2—disagree, 3—indifferent, 4—agree, 5—strongly agree. The evaluation items and the answers are presented in Table 6 and Table 7. The TAM results for the questions related to IndoorPlant utility (Table 6) showed that 63% of users strongly agreed that real-time monitoring is useful in their routine, while 37% only agreed, as shown in Question 1 of Table 6. Question 2 of Table 6 showed that 25% of users were indifferent as to whether the application is connected to the Internet, 37% agreed, and 38% strongly agreed. Some respondents commented that the fact that the cell phone needs to be connected to the Internet is even better for them because the common cell phone signal is poor in the property, even though it is close to the city.

Question 3 in Table 6 showed that 75% of users strongly agreed that the possibility of IndoorPlant predicting plant growth time is useful for them. The remaining users (25%) also agreed that it is useful in their daily lives. The improvement in crop adjustments that IndoorPlant provides to users had its utility approved by 100% of users (Question 4 in Table 6), with 50% strongly agreeing and the other 50% agreeing with the item.

All users (100%) strongly agreed that it is useful to be able to evaluate the harvest made (Question 5 in Table 6), as IndoorPlant saves the current context of the greenhouse, and it may be that the current context is different from the context programmed in the Greenhouse Controller. The possibility of having user profiles for plants (Question 6 in Table 6) was assessed as indifferent by 25% of users; however, 62% strongly agreed that it is useful in daily life and 13% also agreed that it is useful. Users who considered the model having profiles for the plants as indifferent reported that they never change the species of plant grown, so it is always the same parameter that is programmed in the Greenhouse Controller.

Regarding evaluation of the application being via chat, 88% of users strongly agreed that it facilitates use because it is like the applications they normally use, and 12% agreed that it facilitates use, as shown in Question 1 of Table 7. All users agreed that the greenhouse indices are easy to understand, with 88% of users strongly agreeing and 12% agreeing, as presented in Question 2 of Table 7. Some users commented that the visualization of the current greenhouse indices was much easier to understand and see than in the Greenhouse Controller itself.

Question 3 in Table 7 showed that 100% of users agreed that the prediction of harvest time is easy to understand. In the same way, in Question 4 of Table 7, 100% of users strongly agreed that the adjustment indications given by the model are easy to understand. Two users (25%) even commented that if these indications had previously existed, they would have avoided losses because they would not work with the wrong indices in crops, as has happened at other times.

Finally, Question 5 in Table 7 showed that 50% of users strongly agreed that the harvest assessment is easy to use and the other 50% agreed that it is easy to assess the harvest. Question 6 in Table 7 showed that 50% of users strongly agreed and 38% agreed that the use of profiles for plants is useful in cultivation. Only 12% considered the question of plant profiles as indifferent; as previously mentioned, some greenhouses are always used to cultivate the same plant species.

**Figure 11:**
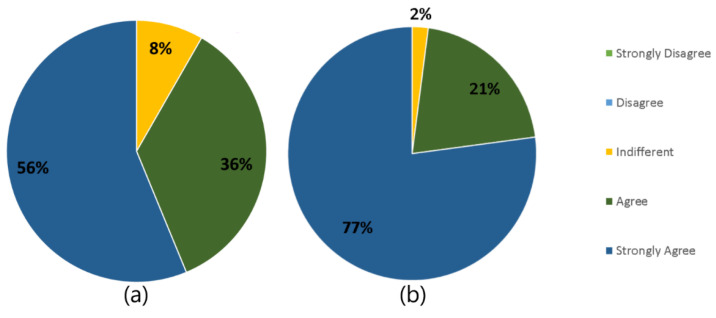
Total utility (**a**) and total ease (**b**) perceived by users.

Analyzing the TAM, it was possible to see that, overall, the proposed model was positively assessed as to utility in 91.66% of the questions, with 56% strongly agreeing and 36% agreeing with its usefulness. Only 8% of the items were rated as indifferent by users, as shown in Figure 11a. Figure 11b shows that in relation to ease of use, only 2% of the model was rated as indifferent, while the other 98% of items were rated positively by users. Of these positively assessed items, 21% of users agreed with the items and 77% strongly agreed that the model’s features are easy to understand and useful.

### 4.3. Discussion of Results

This section presents three scenarios for validation of a model, which serves as a support to farmers for indoor cultivation. In these three scenarios, the services of similarity of greenhouse contexts, management of plant profiles, and prediction of plant productivity were tested. The three scenarios were used the context histories as a basis to suggest improvements, show the existing profiles, and predict the productivity of the cultivation. The scenarios used a Telegram bot for validation by the farmer and communication with the IndoorServer.

By using historical contexts, it was possible to provide services that respond to requests, through the improvements that the model suggests, the display and selection of existing profiles, and the productivity of cultivation. The three scenarios were tested on a network external to the IndoorServer and obtained consistent responses from the server. It was concluded that the IndoorServer met the requests in full. The server supported the receipt of data from three greenhouses for seven months and stored them correctly in their databases.

The results for the first two scenarios were satisfactory because the responses generated and the results obtained were consistent with the reality of the farmers and with the operation, without any unforeseen problems with implementation of the model. In the third scenario, IndoorPlant predicted certain times for each plant to reach the size indicated for harvest and the results had small divergences, as shown in Table 3.

This error was not a cause for concern, as IndoorPlant managed to obtain only seven months of cultivation data as previously mentioned, which also showed that the model did not have all seasons in its context histories. Thus, the error of one or two days in the prediction of cultivation time is tolerable. Table 3 shows that the actual data and the predicted data are close, confirming that the model was successful in predicting the plant cultivation time. It is also important to consider that during data collection, the cultivation time was provided manually by the farmer, which could also cause a small error of information because these data are not obtained automatically.

Based on the behavior presented by the Telegram bot, the IndoorServer, and its application scenarios, it is possible to conclude that the prototype meets what was specified in the model, by comparing and storing context histories, using histories to generate suggestions for improvements to cultivation, and providing the best profiles for the cultivation of each plant, and productivity for the three cultures controlled by Cultiva Fácil Hidroponia.

Through the TAM assessment, it was possible to verify approval of 92% of the items referring to the model’s usefulness. As for the ease of use perceived by TAM, the model obtained 98% approval. Along with these approvals, users suggested some improvements, so it is possible to see some points to be adjusted in IndoorPlant. One of the TAM factors that received great approval from users was the question of the application being in the form of text, thus facilitating its use for those who not regular smartphone users are.

## 5. Final Considerations

This article presented IndoorPlant, a model focused on indoor agriculture that supports intelligent services and addresses the main problems involving agricultural systems that use prediction and/or context sensitivity. The objective of the model is to use context histories to provide a system that helps farmers and provides intelligent services for indoor crops, such as suggestions for improvement, warnings, alerts to problems in cultivation, and calculation of productivity. The proposed infrastructure provides support by monitoring the greenhouse contexts, indicating parameters related to the plant profiles.

In addition, the model supports use of the information generated to make predictions. The model considers user profiles, and monitors and manages the greenhouses, making the information available through the IndoorServer and the Telegram bot. Ubiquitous computing, context sensitivity, and prediction are the starting points for the proposed model; it integrates other technologies, including the ability to add other devices such as different greenhouse controllers that have other sensors, and new predictions that the model can make. This last section presents the main conclusions and contributions of the work, as well as suggestions for future work on this topic.

Section 2 presented the works related to IndoorPlant, as well as a list of criteria to compare them. These other works did not use predictive techniques for productivity but did predict other information about cultivation. Among the information provided are the temperature and humidity of the greenhouse [19]. Studies were also found focusing on decreasing water consumption and intelligent crop irrigation [20,23].

Section 3 described the IndoorPlant model together with its overview, architecture, requirements, and the proposed ontology called Agrindoor. Section 4 presented the implementation and evaluation aspects of IndoorPlant, detailing the technologies applied in the IndoorServer, Telegram bot, Greenhouse Controller (Cultiva Fácil Hidroponia), and three model evaluation scenarios. The three scenarios used data sets of real hydroponic crops, which shows the real capacity of the model with data and everyday situations of agriculture. The prototype showed relevant results when predicting the cultivation time for the three greenhouses and provided the farmer with support and recommendation services that considered the context histories and the profiles saved in the databases.

The application of a questionnaire using the TAM model served to collect usability data from the model indicating that it was well accepted by users. In addition, suggestions for improvements made by the farmers themselves already show the acceptance of the proposed model. These suggestions also show their interest in using technology to facilitate the daily routine.

However, a limitation of the usability assessment must be highlighted. The experiment was restricted to a small group of greenhouse users, due to the focus on the implementation and application of a complete and functional solution based on the proposed model. Additional assessments involving a larger group of users will be needed to confirm and generalize the findings.

One of IndoorPlant’s main contributions is the use of context histories to generate intelligent services with the most varied use. One of these intelligent services tested by the model was productivity prediction, not found in other studies. The use of context histories, context sensitivity, and similarity analysis served to generate suggestions for indoor crops. Other contributions of the model are diversification of the types of cultivation supported by the model (supporting all indoor crops) and the creation of profiles linked to the harvest notes for later use of the same profiles in subsequent crops. In addition to the items compared in Table 1, IndoorPlant is the only study that makes suggestions for improvements in cultivation considering the historical context data.

As future work for IndoorPlant, we will highlight its use in other types of cultivation such as semi-hydroponics. Using other crops will also show IndoorPlant’s potential and ability to monitor, manage, and control more than one crop at a time. Another future work for IndoorPlant is the automation of predictions, where only machine learning training would be manual. This manual work would only be done when it is noticed that the predictions have an error greater than desired. Since sending the current context and obtaining the prediction response can be automated, the prediction would always use the newest and current context of the crops. Using other prediction techniques would also give more value to IndoorPlant and a comparison could be made among the techniques used, to show which one has the best result. The creation of other prediction services can also be cited as future work; after all, the model was designed to support different intelligent services.

The suggestions given by farmers for improvements to the bot are also points to be adjusted to facilitate use and improve the results obtained in their routines. Three users suggested an improvement to the harvest notes service: the addition of intermediate values. As there are only whole values, sometimes the farmer is forced to give a score higher or lower than the true score; if there are notes with differences of 0.5, it would already facilitate evaluation of the harvest. It is also expected to evaluate IndoorPlant with a larger group of farmers and for a longer period, thus allowing a more conclusive assessment of the model’s use.

## Figures and Tables

**Table 1:** Related works.

Article	Sensors	Technique	Plants	Data	Context Histories
Alipio et al. [22]	Air humidity, luminosity, water temperature, pH, and EC	Bayesian networks	Cabbage	Scheduled	No
Goap et al. [20]	UV, soil temperature, soil moisture, soil temperature air, air humidity	Vector regression	Flowers	Scheduled	No
Huong et al. [23]	Soil moisture	Markov’s decision	Tomato	ND	No
Mehra et al. [21]	Tank level, pH, air temperature, air humidity, luminosity	Neural networks	Tomato	Real time	No
Santos et al. [19]	Air temperature, air temperature soil, air humidity, soil moisture, luminosity	ARIMA	Arugula	Scheduled	No
Sisyanto et al. [8]	Air humidity, air temperature, and luminosity	ND	ND	Real time	No
Ni et al. [24]	UAV, accelerometer, tachometer, and anemometer	Vector regression and neural networks	All plants	Scheduled	No
Rossi at al. [26]	Soil humidity, soil temperature, and luminosity	ND	All plants	Real time	No
Brunelli et al. [27]	Light intensity, soil moisture	ND	All plants	Real time	No
Sartori and Brunelli [28]	Phreatimeter	ND	ND	Real time	No
Brunelli et al. [29,31]	Camera	Neural networks	Apple	Real time	No
Segalla et al. [30]	Camera	Neural networks	Apple	Real time	No
IndoorPlant	Air temperature, air humidity, brightness, EC, pH, tank level, amount of nutrients, pump pressure	Analysis of similarity and prediction PLS	All plants	Real time	Yes

**Table 2:** Historical contexts of information obtained.

Property	Format	Description
Air temperature	Decimal	Value refers to the relative temperature in the greenhouse
Air humidity	Decimal	Value for relative humidity in the greenhouse
Soil moisture	Decimal	Value refers to cultivated soil moisture
Water temperature	Decimal	Value refers to circulating water temperature
Water pH	Decimal	Value refers to the circulating water pH
Electrical conductivity	Decimal	Value refers to the electrical conductivity of circulating water
Luminosity	Decimal	Value refers to the amount of light in the greenhouse
Pressure environment	Decimal	Value refers to atmospheric pressure inside the greenhouse
Tank level	Decimal	Value refers to the water level inside the tank
Nutrients	Decimal	Value refers to the nutrients added to the water
Carbon gas	Decimal	Value refers to the percentage of carbon dioxide inside the greenhouse
Pump pressure	Boolean	Value refers to the condition of the circulation pipe
Event	Decimal	Stores current date and time
ID	Decimal	Stores the ID of the greenhouse that sent the data

**Table 3:** Cultivation time for each plant.

Plant	Minimum Time	Maximum Time	Expected Days	Real Days
Radicchio	16	29	21	20
Lettuce	28	44	31	31
Arugula	21	32	25	26

**Table 4:** Prediction results.

Plants	R^2^	RMSEC	RMSECV
Radicchio	0.96	1.06	1.94
Lettuce	0.95	1.37	3.31
Arugula	0.93	1.10	1.89

**Table 5:** Profiles of those who used and evaluated IndoorPlant.

Age	Schooling	Function
40	Elementary school	Radicchio and cabbage farmer
17	Elementary school	Lettuce and broccoli farmer
19	Agricultural technician	Responsible for seedlings
34	Elementary school	Lettuce and arugula farmer
30	Elementary school	Lettuce and arugula farmer
49	Elementary school	Radicchio and cabbage farmer
52	Agricultural technician	Business owner and farmer
20	High school	Radicchio and cabbage farmer

**Table 6:** Items related to assessment of the perceived utility of IndoorPlant.

Question	Description	Answers
1	The ability to track indices in real time is useful	4–37%, 5–63%
2	The possibility of using the application connected to the Internet is useful	3–25%, 4–37%, 5–38%
3	Cultivation time prediction features are useful for the cultivation routine	4–25%, 5–75%
4	The indication of improvements is useful for the cultivation routine	4–50%, 5–50%
5	The notes (classification of the harvest) and profiles services are useful for the cultivation routine	5–100%
6	The use of profiles is useful for the cultivation routine	3–25%, 4–13%, 5–62%

**Table 7:** Items related to assessment of the perceived ease of use of IndoorPlant.

Question	Description	Answers
1	The application being via chat is easy to handle and understand	4–12%, 5–88%
2	The greenhouse indices shown are easy to understand	4–12%, 5–88%
3	The prediction of harvest time is easy to understand	5–100%
4	The indications for adjustments to improve the crop are easy to understand	5–100%
5	Harvest assessment is easy to understand	4–50%, 5–50%
6	Existing profiles and their choices are easy to understand	3–12%, 4–50%, 5–38%

## Abbreviations

The following abbreviations are used in this manuscript:

GSM	Global System for Mobile
GPRS	General Packet Radio Services
ICT	Information and Communication Technology
LoRa	Long Range
MQTT	Message Queuing Telemetry Transport
PLS	Partial Least Square
PMFC	Plant-Microbial Fuel Cell
R^2^	Determination Coefficient
RMSEC	Root Mean Square Error of Calibration
RMSECV	Root Mean Square Error of Cross Validation
TAM	Technology Acceptance Model
UAV	Unmanned Aerial Vehicle
UML	Unified Modeling Language
